# An explanation for the low proportion of tuberculosis that results from transmission between household and known social contacts

**DOI:** 10.1038/s41598-018-23797-2

**Published:** 2018-03-29

**Authors:** Nicky McCreesh, Richard G. White

**Affiliations:** London School of Hygiene and Tropical Medicine, London, UK

## Abstract

We currently have little idea where *Mycobacterium tuberculosis* (*Mtb*) transmission occurs in high incidence settings. Molecular studies suggest that only around 8–19% of transmission to adults occurs within-household, or between known social-contacts. This contrasts with findings from social-contact studies, which show that substantial proportions of contact time occur in households, workplaces and schools. A mathematical model of social-contact behaviour and *Mtb* transmission was developed, incorporating variation in susceptibility and infectiousness. Three types of contact were simulated: household, repeated (individuals outside household contacted repeatedly with daily-monthly frequency) and non-repeated. The model was parameterised using data from Cape Town, South Africa, on mean and variance in contact numbers and contact durations, by contact type, and fitted to an estimate of overdispersion in numbers of secondary cases (‘superspreading’) in Cape Town. Household, repeated, and non-repeated contacts contributed 36%, 13%, and 51% of contact time, and 13%, 8%, and 79% of disease, respectively. Results suggest contact saturation, exacerbated by long disease durations and superspreading, cause the high proportion of transmission between non-repeated contacts. Household and social-contact tracing is therefore unlikely to reach most tuberculosis cases. A better understanding of transmission locations, and methods to identify superspreaders, are urgently required to improve tuberculosis prevention strategies.

## Introduction

We currently have little idea where *Mycobacterium tuberculosis* (*Mtb*) transmission occurs in high incidence settings. A number of studies have used molecular data, combined with household address and/or contact data, to estimate the proportion of all tuberculosis cases attributable to recent within-community transmission that can be attributed to household transmission, or to transmission between known social contacts. In one community in Cape Town, it was estimated that only 8% of (within-community transmitted) tuberculosis resulted from transmission between household members^1^. In another, during the early years of the South African HIV epidemic, 19% of clustered tuberculosis cases could be attributed to household transmission^2^. Elsewhere in South Africa, in KwaZulu-Natal, ‘prolonged or intimate contact’ was identified between only 12% of molecularly linked cases, and casual acquaintanceship between a further 5%^3^. Finally, in Karonga District, Malawi, only 9.4% of all cases in the community could be attributed to transmission from known contacts - 8.2% family, and 1.2% other known contacts^4^.

This contrasts with findings from social-contact studies, which show that substantial proportions of contacts and contact time occur in households. One study reported that 51% and 73% of close contacts (contacts involving skin-to-skin contact and/or conversation) occurred in respondents’ own households in Zambia and South Africa respectively^5^. Another study in South Africa found that 34% of close contacts and 20% of all indoor contacts occurred within respondents’ own households^6^. Findings are similar outside Southern Africa: 18–32% of close contacts in eight European countries^7^ and 85% of close contacts in Vietnam^8^ occurred in respondents’ own homes.

One potential explanation for this disparity is contact saturation – once a household contact has developed disease, then any further infectious contacts with them before the disease is resolved are ‘wasted’^9^. Latent *M.tb* infection also provides some protection from reinfection, leading to an additional source of (partially) wasted household contacts. With variation in infectiousness, a higher proportion of people will not cause any secondary infections, reducing the pool of household contacts available to be infected. Variation in susceptibility will increase this effect, due to the low risk of transmission between people with only moderately infectious tuberculosis and their low-susceptibility household contacts. We therefore hypothesised that this effect of contact saturation will be further exacerbated by variation between people in infectiousness, and in susceptibility to infection and disease progression.

In this paper, we used an individual-based model to simulate patterns of meetings and *Mtb* transmission between three different types of contact: household, regular (met at least once a month), and non-regular (met less frequently, or present at the same indoor location but did not speak). We defined meetings where conversation occurred as ‘close contacts’. This included all household and regular, and some non-regular contacts. We defined meetings that included shared indoor space, but no conversation, as ‘casual contacts’. The model was parameterised using empirical data on social contacts and epidemiology from Cape Town, South Africa^5,10^.

In one set of scenarios, we simulated no variation between people in infectiousness or susceptibility to tuberculosis (beyond that resulting from extrapulmonary, smear positive, or smear negative disease type, and HIV status). In a second set of scenarios, we simulated realistic levels of individual-level variation in infectiousness and susceptibility, by fitting the model to an empirical estimate of overdispersion in the number of secondary cases generated by each person with pulmonary tuberculosis (‘superspreading’). In line with other studies^11,12^, we quantified this by describing the heterogeneity in the number of secondary cases attributable to each infectious person as a binomial distribution. The dispersion parameter (*k*) gives an estimate of the degree of ‘superspreading’. Using molecular data from tuberculosis cases in Cape Town^13^, we estimated k to be 0.15 (95% confidence interval 0.074–0.32).

It is plausible that transmission rates are lower between contacts who do not talk (casual contacts), than those that do (close contacts). For this reason, each set of scenarios consists of three scenarios, in which we assumed the transmission rate between casual contacts was 100%, 50%, and 20% of the transmission rate between close contacts. We refer to these scenarios as high, medium, and low non-repeated casual transmission risk, respectively.

For each scenario, we calculated the proportion of disease that can be attributed to transmission between household, regular, and non-regular contacts, to determine whether the effects of contact saturation (with or without superspreading) were sufficient to explain why the majority of tuberculosis results from transmission outside household and close social contacts in South Africa and other high incidence settings.

## Results

### Model fit to data

**Figure 1:**
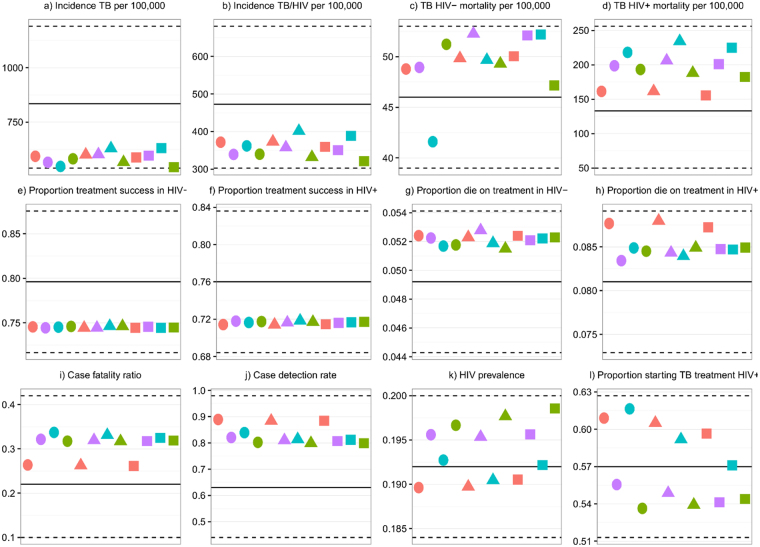
Model fit to data. The solid horizontal lines indicate the best estimates of the output values. The dashed horizontal lines indicate the minimum and maximum of the output plausible ranges. Circles, triangles, and squares indicate the high, medium, and low non-repeated casual transmission risk scenarios respectively. Red indicates scenarios with no additional variation in infectiousness or susceptibility simulated, and purple, turquoise, and green indicate the best estimate of *k*, and lower and upper bounds of the 95% confidence interval for *k* respectively. Full details of each output and plausible range justification are given in the supplementary information.

The model was a good fit to empirical data (Fig. 1). The value of the dispersion parameter *k* (a measure of the degree of overdispersion) was within ±1% (relative) of the target value in all scenarios.

### Proportion of transmission

**Figure 2:**
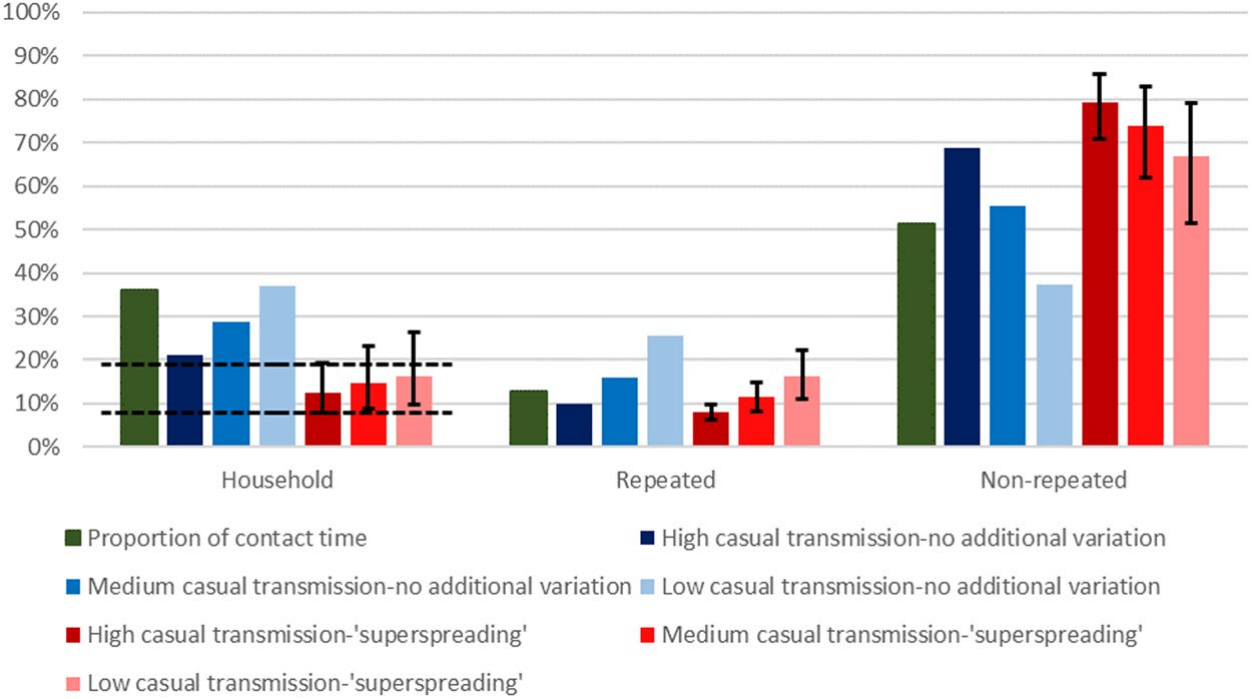
Proportion of contact time, and proportion of disease resulting from transmission between household, repeated, and non-repeated contacts with no additional variation in susceptibility and infectiousness, or with ‘superspreading’, in the high, medium and low casual transmission scenarios. For the ‘superspreading’ scenarios, coloured bars show results for the best estimate of the dispersion parameter *k*. Error bars indicate results for the upper and lower bounds of the 95% confidence intervals for *k*. Horizontal dotted lines show the range of the proportion of tuberculosis estimated to result from household transmission in empirical studies in sub-Saharan Africa^1–4^.

If we assume that the proportion of disease attributable to each type of contact is proportional to meeting time, then an estimated 36.0%, 12.7%, and 51.3% of disease would result from transmission between household, repeated, and non-repeated contacts respectively (Fig. 2, green bar).

When we simulated patterns of meetings and transmission, assuming no additional variation in infectiousness or susceptibility, the proportion of tuberculosis cases resulting from transmission between household members in the model was 21.2%, 28.6%, and 37.1% in the high, medium, and low non-repeated casual transmission risk scenarios respectively (Fig. 2), higher than the 8–19% suggested by empirical data. The overall proportion resulting from transmission between repeated contacts was 10.1%, 16.0%, and 25.7%, respectively. The overall proportion resulting from transmission between non-repeated contacts was 68.7%, 55.4%, and 37.2%, respectively.

When the model was fitted to the empirical estimates of ‘superspreading’ (*k* = 0.15 (*k* = 0.074 – *k* = 0.32)), the proportion of disease resulting from transmission between household members was 12.6% (7.87%-19.3%), 14.6% (8.78%-23.2%), and 16.5% (9.87%-26.4%) in the high, medium, and low non-repeated casual transmission risk scenarios respectively, consistent with the 8–19% suggested by empirical data (Fig. 2). The proportion of tuberculosis cases resulting from transmission between repeated contacts was 8.16% (6.24%-9.90%), 11.6% (8.30%-14.8%), and 16.5% (11.1%-22.2%) in the high, medium, and low non-repeated casual transmission risk scenarios respectively. Of this, 19.8%-38.8% was between contacts who see each other daily (derivable from data in Table 1). The proportion tuberculosis cases resulting from transmission between non-repeated contacts was 79.3% (85.9%-70.8%), 73.8% (82.9%-62.0%), and 67.0% (79.0%-51.4%) in the high, medium, and low non-repeated casual transmission risk scenarios respectively. The majority of this (93.7–98.7%) was between contacts who did not talk (derivable from data in Table 1).

**Table 1:** Proportion of contacts and meeting time, and proportion of tuberculosis cases resulting from transmission between household, repeated, and non-repeated contacts.

		Empirical data		Model results, no variation in susceptibility or infectiousness			Model results, fitted to empirical estimate of k (95% confidence interval for k)		
		Proportion of contacts (per day)	Proportion of meeting time	High casual transmission	Medium casual transmission	Low casual transmission	High casual transmission	Medium casual transmission	Low casual transmission
Household		20.4%	36.0%	21.2%	28.6%	37.1%	12.6% (7.87%-19.3%)	14.6% (8.78%-23.2%)	16.5% (9.87%-26.4%)
Repeated	Daily	5.14%	5.76%	3.99%	5.92%	8.68%	2.51% (1.59%-3.68%)	3.12% (1.85%-4.85%)	3.81% (2.20%-6.11%)
	Weekly-monthly	8.12%	6.82%	6.15%	10.1%	17.1%	5.65% (4.65%-6.22%)	8.48% (6.46%-9.92%)	12.7% (8.92%-16.1%)
	Overall	13.3%	12.7%	10.1%	16.0%	25.7%	8.16% (6.24%-9.90%)	11.6% (8.30%-14.8%)	16.5% (11.1%-22.2%)
Non-repeated	Non-repeated close	1.21%	0.678%	0.918%	1.45%	2.36%	1.06% (1.14%-0.95%)	1.92% (2.16%-1.61%)	4.11% (4.60%-3.22%)
	Non-repeated casual	63.5%	49.4%	67.8%	54.0%	34.8%	78.2% (84.8%-69.9%)	71.9% (80.8%-60.4%)	62.9% (74.4%-48.2%)
	Overall	66.4%	51.3%	68.7%	55.4%	37.2%	79.3% (85.9%-70.8%)	73.8% (82.9%-62.0%)	67.0% (79.0%-51.4%)

**Figure 3:**
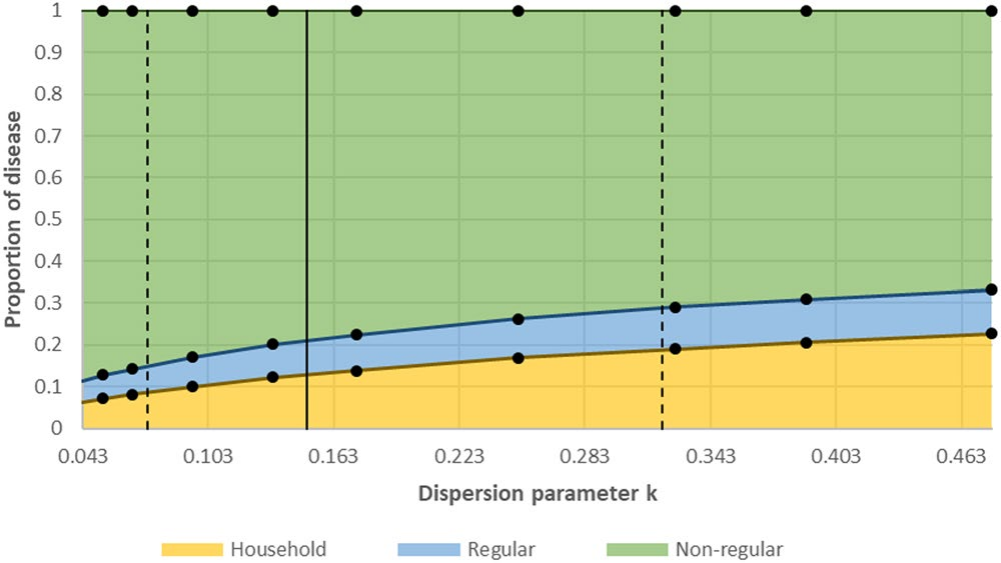
The proportion of disease resulting from transmission between household, regular, and non-regular contacts, at different values of the dispersion parameter *k*. Dots indicate points where model runs were carried out. The solid and dashed vertical lines show the best empirical estimate of *k*, and the 95% confidence intervals for k, respectively.

As ‘superspreading’ was reduced (i.e. *k* was increased), the proportion of disease attributable to transmission between household and regular contacts increased, and the proportion of disease attributable to transmission between non-regular contacts decreased (Fig. 3).

### Sensitivity analyses

Our results were robust to sensitivity analysis. There was little variation between the baseline scenario and the sensitivity analyses scenarios in the proportion of disease resulting from transmission between household members (12.6% in baseline, 11.6%-13.4% in sensitivity analyses), repeated (8.16% in baseline, 5.97%-12.5% in sensitivity analyses), or non-repeated (79.3% in baseline, 74.6%-81.3% in sensitivity analyses) contacts. Full results are given in the supplementary information.

### Overdispersion

**Figure 4:**
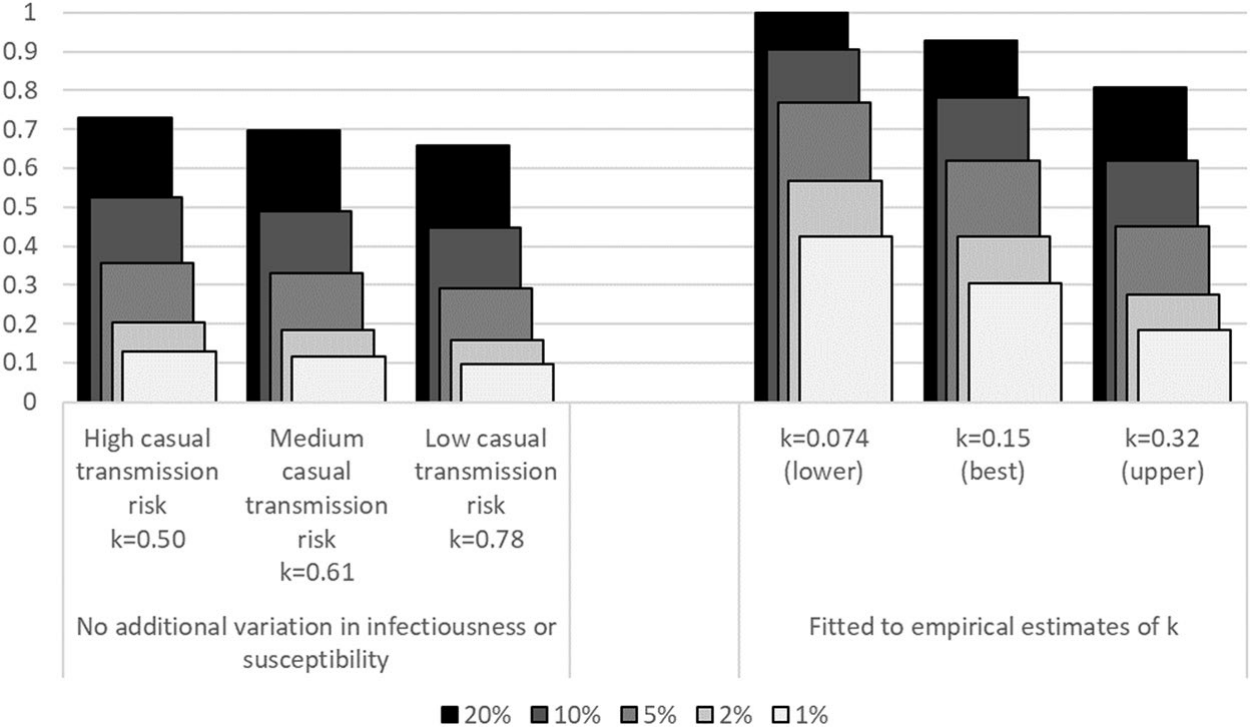
Proportion of tuberculosis cases resulting from transmission by the most highly transmitting 20%, 10%, 5%, 2% or 1% of people with pulmonary tuberculosis. Bars on the left show the results from scenarios with no additional variation in infectiousness or susceptibility. Bars on the right show the results from scenarios where the model was fitted to empirical estimates of *k*, in the high casual transmission risk scenarios. The numbers below the bars show the dispersion parameter, *k*, from fitting a negative binomial distribution to the number of tuberculosis cases resulting from transmission by each person with pulmonary tuberculosis. Results for the medium and low casual transmission risk scenarios where the model was fitted to estimates of *k* were very similar to those for the high casual transmission risk scenario (right bars), and were therefore not shown.

In the scenarios where no additional variation in infectiousness or susceptibility was simulated, in the three casual transmission risk scenarios, between 66–73% of disease resulted from transmission by the top 20% of transmitters, 45–52% from the top 10%, 29–36% from the top 5%, 16–20% from the top 2%, and 10–13% from the top 1% (Fig. 4).

When the model was fitted to the lower, best and upper empirical estimates of *k*, in the high casual transmission risk scenarios, 100%, 93%, and 81% of disease resulted from transmission by the top 20% of transmitters respectively; 90%, 78%, and 62% by the top 10%; 77%, 62%, and 45% by the top 5%; 57%, 42%, and 28% by the top 2%; and 42%, 30%, and 18% by the top 1% (Fig. 4). Results for the medium and low casual transmission risk scenarios were very similar to those for the high casual transmission risk scenario, and are therefore not shown.

## Discussion

Studies of social contacts suggest that large proportions of indoor meeting time between people occur between household members or close social contacts. Despite this, analysis of molecular data from tuberculosis cases indicates that transmission between household members is unlikely to be responsible for more than a small proportion of disease in high incidence settings. In this paper, we provide an explanation for this disparity.

In our setting, meetings between household members were responsible for 36% of adult meeting time. When contact patterns in the community were simulated, assuming no variation in susceptibility or infectiousness (beyond those due to HIV status and disease type) and no reduction in transmission probabilities between contacts who do not talk, household transmission was estimated to be responsible for around 21% of disease. This demonstrates the effects of contact saturation, in the absence of large amounts of overdispersion in number of secondary cases. Simulating greater overdispersion, ‘superspreading’, exacerbated the effects of contact saturation, and reduced the proportion of disease resulting from household transmission to 13% (7.9–19%), in line with the estimates from molecular studies (8–19%).

Our findings have implications for tuberculosis care and control in high incidence settings. With only a small proportion of tuberculosis resulting from household and close social contact transmission, contact tracing in households or among close contacts is unlikely to have a large effect on the incidence of tuberculosis at a community level. This is supported by empirical studies that identify few epidemiological links between molecularly clustered tuberculosis cases^14,15^. Our results suggest that the majority of disease results from infection by a small proportion of people with tuberculosis, often referred to as superspreaders. These people are likely to combine high infectiousness (e.g. generation of culture positive cough aerosols^16^) and/or long durations of infectious, with high numbers of contacts (for instance due to employment in busy congregate settings). This highlights a critical need to develop new technologies and strategies to reliably identify tuberculosis superspreaders. Once developed, targeting case-finding at congregate settings regularly visited by superspreaders may become a high impact, and potentially highly cost-effective, intervention.

Our findings also have implications for the collection of social contact data for understanding infection transmission, in particular for infections with long durations and/or superspreading characteristics, such as *M.tb*. Many social contact studies collect data on contacts that were spoken to or touched only, neglecting contacts that were merely present in the same indoor location at the same time^7,17–20^. In our study setting, these casual contacts were responsible for 64% of contacts and 49% of meeting time, and a disproportionately large proportion of tuberculosis – 89%, 74%, and 67%, assuming that the transmission probability between casual contacts was 100%, 50%, and 20% of that between other contacts respectively.

Our study has a number of limitations. Our estimate of the overdispersion parameter *k* for Cape Town may be under- or overestimated. Restriction fragment length polymorphism (RFLP) data were only available from an estimated 27% of people with pulmonary tuberculosis in the molecular study from Cape Town^13^. While we adjusted for this in estimating *k*, we assumed that the probability of a person with pulmonary tuberculosis being missing from our data was unrelated to infectiousness. In practice, people with low infectiousness may be more likely to be missing from our sample, due to under-diagnosis or missing RFLP data. This may have resulted in us underestimating the true number of non-clustered cases, and overestimating *k* as a result (i.e. underestimating superspreading). Alternatively, immigration into the community may have resulted in us overestimating non-clustered cases, and underestimating *k*. There is also a large amount of uncertainty in the proportion of data missing, due to uncertainty in the proportion of people with tuberculosis who are diagnosed^21^, however this has little effect on estimates of *k* (*k* = 0.12 (0.060–0.27) and 0.18 (0.094–0.37) with 44% and 99% case detection respectively). A full discussion of the limitations in the data and methods used in estimating *k* are given in Middelkoop *et al*.^13^ and Ypma *et al*.^12^ respectively.

We limited our model to people aged 15+ years, as detailed contact data from children were not available. This will have little effect on our estimates of transmission between different contact types in adults, as the risk of transmission from children is low^22^. The contribution of household contact to tuberculosis in children is likely to be higher than for adults however^23^, and therefore our findings do not apply to children. The proportion of transmission between different types of contact may also be different for other settings, although data from molecular studies consistently show low household transmission in high incidence settings^1–4^.

There are likely to be inaccuracies in our estimates of contact numbers and meeting durations in Cape Town, due to the challenges in the collection of accurate social contact data. For instance, numbers of non-household contacts spoken to may be underestimated, due to recall bias or questionnaire fatigue; and numbers of non-repeated casual contacts may be under- or over-estimated, due to recall bias and difficulties in estimating the number of people present in a location. The relative contribution of non-repeated vs repeated contacts to overall tuberculosis in our model may therefore be over- or underestimated. It is also likely that some repetition of non-conversational, casual contacts occurs in real life, which were not captured in the model, however sensitivity analysis shows that this is likely to have little effect on our conclusions (see supplementary information). Similarly, there are difficulties in accurately estimating the proportion of transmission that occurs within households or between close social contacts from molecular data^1^.

To conclude, contact saturation, exacerbated by superspreading, may be why only a small proportion of tuberculosis results from household transmission in high incidence settings. This has implications for tuberculosis control. Even if yields are high, household and social-contact tracing is unlikely to reach most tuberculosis cases. A better understanding of transmission locations, and methods for identifying superspreaders, would assist in the design of higher impact tuberculosis control strategies.

## Methods

### Contact data

In the social contact literature, ‘contact’ is used both as a noun to refer to the people met, and as a verb to refer to the meeting or shared space between people. For clarity, in this paper, we reserve the term ‘contact’ to refer to people met, and use the term ‘meeting’ to refer to people sharing indoor space.

Estimates of social contact meeting time were taken from a survey conducted in eight communities in Western Cape, South Africa in 2011^5,10^. 1270 adults aged 18+ years were interviewed using a structured questionnaire. Contact data were collected using four methods:

1. Respondents were asked for basic demographic information on their household members
2. Respondents were asked for details of all people with whom they had a face-to-face conversation the day preceding the interview
3. Respondents were asked to give information on all buildings (other than their own home) they went into on the day preceding the interview, including estimates of numbers of people present
4. Respondents were asked questions about minibus use, and their last minibus journey

From these data, it was possible to estimate mean and variance in numbers of contacts aged ≥12 years seen the day preceding the interview, and mean meeting durations, for five types of contact. In line with other studies, we define close contacts as contacts with whom the respondent had a face-to-face conversation, and casual contacts as contacts with whom the respondent shared indoor space, without conversation^5^. Contacts can be grouped into three categories:

1. **Household (Repeated, Close, Household)**. Contacts who are members of the same household
2. **Repeated (Repeated, Close, Non-Household)**. Individuals (not from the respondent’s own household) spoken to, seen with at least monthly frequency. These are split into:
  - **Daily**. Contacts that respondents report that they see with daily frequency
  - **Weekly-monthly** Contacts that respondents report that they see ‘1–6 times a week or ‘1–3 times a month’
3. **Non-repeated (Non-repeated, Close or Casual)**. Individuals seen with less than monthly frequency, or never seen before. These are split into:
  - **Non-repeated close**. Contacts *spoken to* that respondents report that they see ‘less than monthly’, or ‘never before’
  - **Non-repeated casual**. Contacts present in the same indoor location (building or minibus), but not spoken to

Table 2 shows the mean and variance in daily contact numbers, and meeting durations, by contact type. Full details of the contact data are given in the supplementary information.

**Table 2:** Empirical estimates of contact numbers and meeting durations, by contact type. Only contacts aged 12+ years were included in the analysis. Underlining indicates figures used directly as model input parameters.

Contact type		Mean contacts/person/day	Variance in contacts/person/day	Mean meeting duration (minutes)	Mean meeting time/person/day (hours)
Household		2.46	2.24	415	17.0
Repeated	Daily	0.62	1.41	263	2.72
	Weekly-monthly	0.98	1.93	197	3.22
	Overall	1.60	2.66	225	6.00
	*Proportion of repeated contacts that are daily*			*0.387*	
Non-repeated	Non-repeated close	0.146	0.435	132	0.32
	Non-repeated casual	7.67	95.0	182	23.3
	Overall	8.01	95.7	181	24.2
	*Proportion of non-repeated contacts that are non-repeated close*			*0.0187*	
Total		12.07	110	235	47.2

### Model description

We used an individual-based model, coded in Netlogo 5.3.1^24^. The model had a fixed population size of 20,000 people aged 15–60. People aged <15 years were not simulated, as the risk of transmission from children is low^22^, and detailed contact data were not available from children. The model simulated *Mtb* transmission; disease development, mortality, treatment and cure; and HIV and its effects on tuberculosis.

Five types of social contact were simulated, corresponding to the five types of contact outlined above. As before, these can be grouped into three categories:

1. **Household**. Each simulated individual was assigned to a household. Household size and membership was fixed, only changing when a member died and was replaced.
2. **Repeated**. Each individual was assigned a fixed number of repeated contacts. These contacts were fixed, only changing when a contact died and was replaced. Each repeated contact was assigned to one of two types:
  - **Daily**. Daily contacts were met every day.
  - **Weekly-monthly**. Simulated individuals had a pool of contacts of this type 5 times the size of the average number they meet each day. Each weekly-monthly contact was met each day with a probability 0.2.
3. **Non-repeated**. Each individual was assigned a fixed number of non-repeated contacts to meet each day. These were chosen at random each day, from all people in the model, with the selection probability proportional to each individual’s own number of non-repeated contacts. Each time a meeting occurred, it was assigned at random to one of two types:
  - **Non-repeated close**. Meetings which included conversation
  - **Non-repeated casual**. Meetings between people present in the same indoor location or minibus, but where conversation did not occur

For all five contact types, the model was parameterised using empirical data on the mean and variance in contact numbers of that type seen by each person per day. Meeting durations for each type of contact were fixed, and were set equal to the mean estimates from the empirical data. In line with the empirical contact data, conversation was assumed to occur between all types of contact except non-repeated casual contacts. Table 3 summarises the simulated contact types. A diagram showing how the contact data links to the model contact structure and contact input parameters is given in the supplementary information.

**Table 3:** Model contact types and characteristics. *The relative transmission probability for non-repeated casual contacts was assumed to be 1, 0.5, and 0.2 in the high, medium, and low non-repeated casual transmission risk scenarios respectively.

Contact type		Simulated as:	Mean number of contacts	Daily probability of meeting with each contact-person	Mean number of contacts per day	Clustering coefficient	Meeting duration (minutes)	Relative transmission probability
Household		Each person has fixed number of contacts	2.46	1	na	1	415	1
Repeated	Daily	Each person has fixed number of contacts	0.62	1	na	0.2	263	1
	Weekly-monthly	Each person has fixed number of contacts	4.9	0.2	na		197	1
Non-repeated	Non-repeated close	Chosen at random from model population each day	na	na	0.146	na	132	1
	Non-repeated casual	Chosen at random from model population each day	na	na	7.67	na	182	1/0.5/0.2*

Each individual in the model was assumed to have an individual level of susceptibility to *Mtb* infection and disease progression, and an individual level of infectiousness with pulmonary tuberculosis. These were modelled as individual-level susceptibility parameters and infectiousness parameters, and were selected at birth from gamma distributions with mean 1 and variance *susceptibility_‌var* and *infectiousness_‌var* respectively. Gamma distributions were chosen, as gamma distributed infectiousness parameters result in a negative binomial distribution of secondary cases. The *susceptibility* parameter is assumed to incorporate the effects of all risk factors that have an effect on the risk of infection and/or disease development, with the exception of HIV, which is explicitly simulated. It is assumed that 25% of the variation in susceptibility acts through altering susceptibility to infection, and 75% through altering the risk of progression to disease^25^. The effects of this assumption are explored in the sensitivity analysis. The *infectiousness* parameter is assumed to incorporate the effects of all risk factors that have an effect on the infectiousness of a person with tuberculosis, with the exception of whether the disease is pulmonary smear positive, pulmonary smear negative, or extrapulmonary, which is explicitly simulated.

A full description of the model is given in the supplementary information.

### Estimating ‘super-spreading’

In line with other studies^11,12^, we quantify the degree of superspreading by describing the heterogeneity in the number of secondary cases (of pulmonary tuberculosis) attributable to each infectious person (person with pulmonary tuberculosis) as a binomial distribution. The dispersion parameter of the distribution, *k*, indicates the degree of superspreading, with a value of 1 suggesting no over-dispersion, and lower values indicating higher amounts of over-dispersion (superspreading). Using methods published by Ypma *et al*. (2013)^12^, we estimated *k* for Cape Town to be 0.147 (95% CI 0.0737–0.317), using data on tuberculosis cluster sizes between 2001 and 2010 in a peri-urban township of Cape Town^13^. In calculating *k*, we assume that RFLP data were available for 27% of cases (42% × 63%). This is because RFLP data were available from only 42% of diagnosed cases of pulmonary tuberculosis^13^, and it is estimated that only 63% (95% CI 44%-99%) of people with tuberculosis are diagnosed in South Africa^21^. Assuming that 44% and 99% of people tuberculosis are diagnosed, we estimate *k* to be 0.123 (0.0595–0.269) and 0.179 (0.0941–0.370) respectively.

### Model parameterisation and fitting

The model was fitted to WHO estimates for South Africa of overall and HIV-associated tuberculosis incidence, population level mortality rates from tuberculosis in HIV− and HIV+ people, the tuberculosis case detection rate, the proportion of people starting tuberculosis treatment who are HIV+^21^; South African national data on treatment outcomes (success and mortality) in HIV− and HIV+ people^26^; and UNAIDS estimates of adult HIV prevalence^27^. Full details of all fitting outputs are given in the supplementary information.

In one set of scenarios, we simulated no additional variation in infectiousness and susceptibility (beyond that resulting from infection type (extrapulmonary, smear positive, or smear negative), and HIV status). In the second set, we fitted model output on the number of pulmonary disease cases resulting from transmission from each potential transmitter (simulated person with pulmonary disease), to the empirical estimates of *k*: *k* = *0.15* as the best estimate, plus *k* = *0.074* and *k* = *0.32* as upper and lower bounds. This was done by varying the amount of variation in infectiousness and susceptibility.

It is plausible that transmission rates are lower between contacts who do not talk (casual contacts) than those that do. For this reason, each set of scenarios consists of three sub-scenarios, in which we assumed the transmission probability per minute of meeting between casual contacts was 100%, 50%, and 20% of the transmission probability between close contacts; named ‘high’, ‘medium’, and ‘low’ non-repeated casual transmission risks, respectively.

To determine the effects of variation in infectiousness and susceptibility on transmission location, we also simulated scenarios where we varied the level of variation in infectiousness and susceptibility over a wide range. The model input parameter determining the baseline probability of *Mtb* transmission per minute meeting time was varied, to fit the model to the plausible ranges for the calibration targets. In these scenarios, we did not constrain the model output *k*.

The model was run until equilibrium was reached before any results were outputted. All results are averaged over 200 model runs. The proportion of disease resulting from transmission between household, regular, and non-regular contacts was calculated.

### Sensitivity analyses

A number of sensitivity analyses were conducted, exploring the effects of varying the probability that weekly-monthly contacts are seen each day, varying the proportion of variation in susceptibility that acts through altering the risk of infection (as opposed to disease progression), varying the clustering coefficient for repeat contacts, varying the degree of variation in susceptibility relative to the degree of variation in infectiousness, and assuming that some casual contacts were repeated. All sensitivity analyses were conducted using the high non-repeated casual transmission risk scenario, fitted to the best estimate of *k* (*k* = 0.15). They are described in full in the supplementary information.

### Code availability

Model code will be made publicly available from the London School of Hygiene and Tropical Medicine ‘Data Compass’ repository upon publication of the manuscript, with no restrictions.

## Electronic supplementary material

Supplementary information
