# Supplementary material for: An explanation for the low proportion of tuberculosis that results from transmission between household and known social contacts
Source: Sci Rep. 2018 Mar 29;8:5382. doi: 10.1038/s41598-018-23797-2 (PMC5876383; doi:10.1038/s41598-018-23797-2)
Supplement: Supplementary file 1 — Supplementary information [file 41598_2018_23797_MOESM1_ESM.pdf]

An explanation for the low proportion of tuberculosis that results from transmission between household and known social contacts

Supplementary information

Nicky McCreesh[1]* and Richard G White[1]

[1]London School of Hygiene and Tropical Medicine, UK

*corresponding author

Model and data description

Key

Model parameter names are written in italics, with colour indicating whether the parameter is an input parameter, a parameter with a global model-wide value, calculated from input parameter(s), or an individual-level parameter, which can take a different value for each simulated person.

Social contact

Data

Estimates of social contacts and meeting durations were taken from a survey of social contact behaviour conducted in eight communities in Western Cape, South Africa in 2011. 1270 adults aged 18+ years were interviewed using a structured questionnaire. Basic demographic information was collected, and contact data were collected using four methods:

Method 1. Respondents were asked for details of each member of their household, including age

Method 2. Respondents were asked to give details of all people with whom they had a face-to-face conversation the day preceding the interview. For each person, they were asked a number of questions, including:

a. Do they belong to your household?
b. Where were you when you were with them most?
c. What was the total amount of time you were with them during the day?
d. How often do you normally talk to this person?

Method 3. Respondents were asked to list all buildings (other than their own home) they went into on the day preceding the interview. For each building, they were asked a number of questions, including:

a. How much time did you spend in total inside this building?
b. How many adults and youth (those older than 12) were present?

Method 4. Finally, respondents were asked a number of addition questions about certain, selected activities. These included the following questions about minibus use:

a. Do you ever use minibuses?
b. The last time you used a minibus, how many adults and youths (those aged more than 12) were inside the minibus?
c. The last time you used a minibus, how long were you inside the minibus?
d. During the last week, how many times did you use a minibus?

In this study, we define a meeting as being present in an indoor location at the same time, regardless of whether conversation and/or touching occurred. Using the data collected, we distinguish five categories of contact:

1) **Household**. The mean and variance of household size (excluding children aged ≤12 years) in the population were calculated from questions on household members. The mean duration of meeting with household members per day was calculated from data collected using method 1. The duration was then adjusted, to account for the fact that not all household members will be seen or spoken to each day (e.g. due to travel by household members). This was done by multiplying the mean meeting duration by the ratio of the numbers of household members spoken to the preceding day (calculated using the data collected using method 1) to the mean number of household members aged >12 years.
2) **Daily contacts (non-household)**. Non-household contacts occurring in indoor settings collected using method 1 were split into three categories, based on the response to the question 'How often do you normally talk to this person?'. Where the response was 'Daily', contacts were classed as daily contacts. Data on these contacts were used to determine the mean meeting duration with daily contacts.
3) **Weekly-monthly contacts.** Non-household contacts occurring in indoor settings collected using method 1, where the response to the question 'How often do you normally talk to this person?' was '1-6 times a week or '1-3 times a month', were classed as weekly-monthly contacts. Data on daily and weekly-monthly contacts were used to determine the mean and variance in numbers of repeated (daily and weekly-monthly) contacts per day, the proportion of repeated contacts that are daily, and the mean meeting duration with weekly-

monthly contacts. Daily and weekly-monthly contacts were grouped in this way to allow the overall level of clustering between repeated contacts in the model to be controlled, and set at the desired level.

4) **Non-repeated close contacts.** Non-household contacts occurring in indoor settings collected using method 1, where the response to the question ‘How often do you normally talk to this person?’ was ‘Less than monthly’, or ‘Never before’, were classed as non-repeated close contacts. Data on these contacts were used to determine the mean and variance in numbers of non-repeated close contacts per day, and the mean duration of contact per day with non-repeated close contacts.
5) **Non-repeated casual contacts.** Non-repeated casual contacts were defined as people who the respondent was in the same indoor location or minibus with, but with whom they did not speak. Data on these contacts were not collected directly, and therefore information on these contacts were estimated using indirect means. First, the number of total contacts in buildings per day was calculated for each respondent using data collected using method 2, by summing the numbers of adult and children present in each building visited. The number of contacts in minibuses per day was added to this, using data collected using method 3. The total number of contacts that each respondent spoke to per day (contact types 1-4) was then subtracted from this, to give the number of non-repeated casual contacts per day. The mean and variance in the number of non-repeated casual contacts per day could then be calculated. The mean duration of non-repeated casual meetings was calculated as the mean of the time spent on minibus journeys and the time spent in buildings (other than the respondents’ own home), weighted by the number of non-repeated casual contacts present.

The number of non-repeated close and non-repeated casual contacts were summed for each individual, to give the overall number of unrepeated contacts. From this, the mean and variance in the number of unrepeated contacts, and the proportion of unrepeated contacts that involved conversation (non-repeated close contacts) were calculated.

All estimates were weighted to account for the sampling design and the day of the week that participants were asked about (weekend or weekday). As meeting and building visit durations and numbers of people present were recorded categorically, category mid-points were used in calculations (e.g. a duration of 7.5 minutes was used for reported visits of 5-10 minutes). A duration of 14 hours was used for visits that were reported to have lasted more than 14 hours, and a duration of 8 hours for meetings that lasted more than 8 hours. Visits where more than 20 adults/youths were reported to have been present were assigned a value of 30.

Model

Five types of social contact are simulated, corresponding to the five categories of social contact described above. Social contacts remain fixed for the duration of each model run. When an individual dies, they are replaced by another individual, who is assigned exactly the same social contacts (see section Demography for details). There cannot be more than one type of repeated contact (household, daily, or weekly-monthly) between the same two individuals. Figure S1 shows how the contact data links to the model contact structure and contact input parameters.

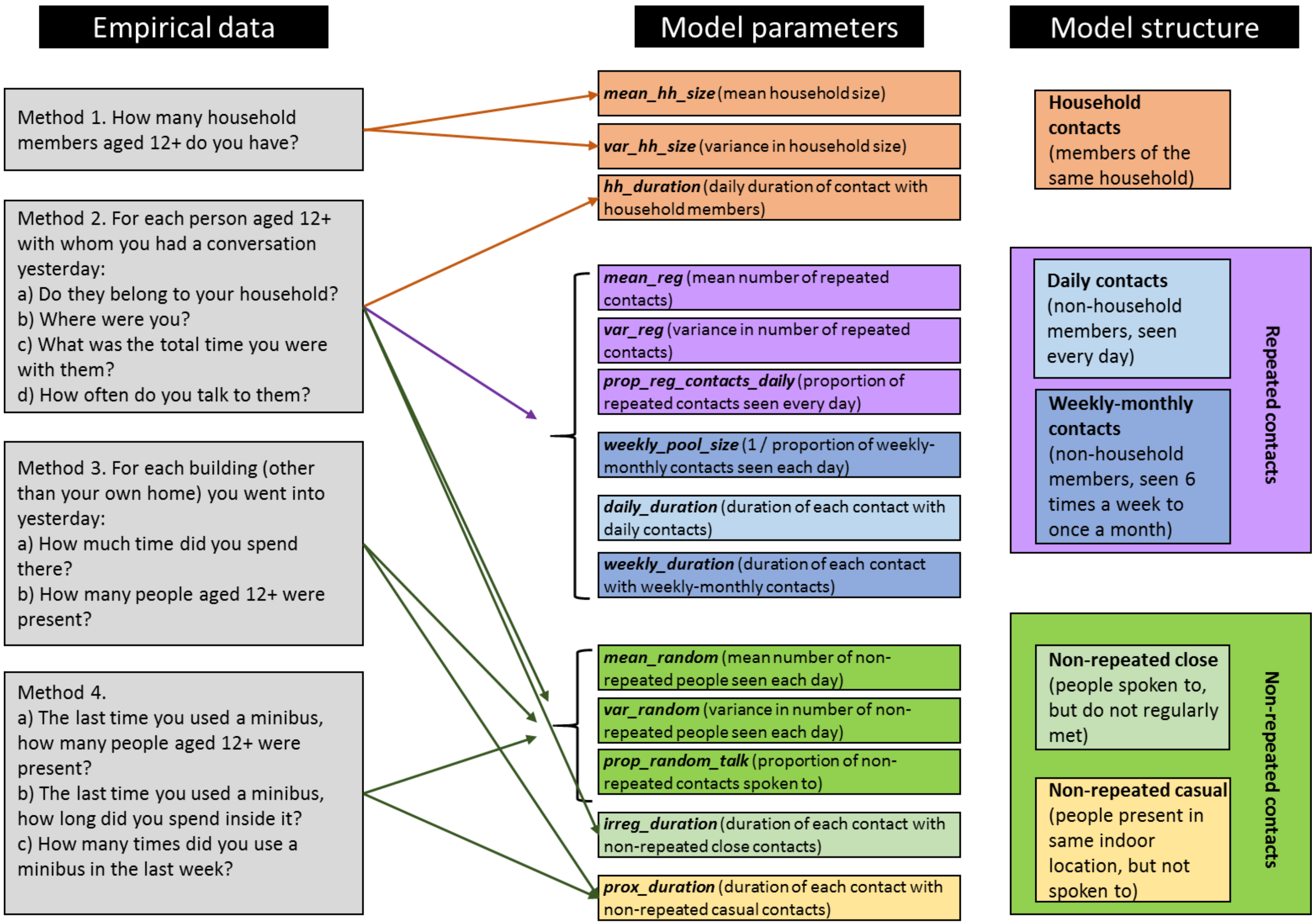

Figure S1. Diagram of contact data, model parameters, and model contact structure.

Household

During model initiation, a list of household sizes is generated by repeatedly sampling numbers from a gamma distribution, with mean *mean_hh_size* and variance *var_hh_size*. Simulated individuals are then assigned to these households at random. The size of each household remains constant over time, and simulated individuals do not change households. Each individual is assumed to have contact with each other member of their household for *hh_duration* per day.

Daily and weekly-monthly (non-household)

For each simulated individual, a provisional repeated contact number, *provisional_reg_contacts,* is selected from a gamma distribution with mean *mean_reg* and variance *var_reg*

The number of weekly-monthly contacts individuals' have was calculated from data on the number of contacts spoken to each day who the respondent reports meeting '1-6 times a week' or '1-3 times a month'. 82% of these contacts were people seen '1-6 times a week'. If an individual reports *N* meetings with contacts of this type, then they could have a total of *7N* contacts, who they meet once a week; a total of *(7/6)N* contacts, who they meet 6 times a week; or anything between these two extremes. Similarly, people seen '1-3 times a month' could consist of many people met more infrequently, or fewer people met less frequently. An input parameter, *weekly_pool_size*, was therefore used to determine the mean total number of weekly-monthly contacts each simulated individual should have.

The provisional repeated contact number for each individual is therefore adjusted, to allow for the higher number of weekly-monthly contacts needed, using the formula:

*reg_contacts = round (provisional_reg_contacts * prop_reg_contacts_daily + provisional_reg_contacts * (1 - prop_reg_contacts_daily) * weekly_pool_size)*

where *prop_reg_contacts_daily* is the overall proportion of repeated contacts seen each day who are met every day.

Now that the number of repeated contacts that each simulated person should have is known, links need to be made between people until everyone has the correct number of contacts, and the model has the desired level of clustering between repeated contacts. This is done as follows:

1) As the total number of repeated contacts people have needs to be divisible by 2, if the sum of *reg_contacts* over all people in the model is odd, then a person with *reg_contacts > 1* is chosen at random, and 1 is subtracted from their *reg_contacts*.

2) The total number of closed triplets needed is calculated using the formula: *round((*$\sum_N (reg\_contacts \times reg\_contacts - 1)$ * *clustering_coefficient),* where N is model population.
3) The model cycles between adding small numbers of links between people at random, and adding links to close triplets. This continues until the desired number of closed triplets has been added, at which point remaining links are added at random. Throughout this process, links can only be added to people who have fewer links than their desired number of repeated contacts (*reg_contacts*), and there cannot be more than one link between the same pair of people
4) Finally, each repeated contact link is made a daily contact with probability *prop_reg_contacts_daily / (prop_reg_contacts_daily + (1 - prop_reg_contacts_daily) * weekly_pool_size)*

   Repeated contact links not chosen to be daily contacts are made weekly-monthly contacts.

Each individual is assumed to meet with each daily contact for *daily_duration* per day. Each individual is assumed to meet with each of their weekly-monthly contacts each day with probability *1 / weekly_pool_size*. When a meeting occurs, it is assumed to last for *weekly_duration*.

Non-repeated close and non-repeated casual contacts

For each simulated individual, a daily non-repeated (non-repeated close and non-repeated casual) contact number is selected from a gamma distribution with mean *mean_random* and variance *var_random*. Each infectious individual is assumed to meet with this number of people a day, selected at random from the whole model population (with the selection probability proportionate to each individual's own number of non-repeated contacts). Each non-repeated contact pair is assumed to talk to each other with probability *prob_random_talk* (non-repeated close contact), and to not talk with probability *1 - prob_random_talk* (non-repeated casual contact). These meetings are assumed to last *irreg_duration* and *prox_duration* respectively.

Model initialisation

To initialise the model, 20,000 people are created, with their ages drawn from a uniform distribution between 15 and 60. Each person is infected with HIV with probability *initial_prop_HIV_pos*, and 200 are given smear positive pulmonary TB.

The initialisation of social contacts is described in the section Social contact – model.

The model is run for 50 years until equilibrium is reached before any results are outputted.

Demography

Individuals are introduced into the model at age 15, and die at age 60. People aged <15 are not modelled, as the risk of *Mtb* transmission for children is low[1], and detailed contact data were not available from children from the population that the adult contact data were from.

The model population size is kept constant: when an individual dies (at age 60 or of TB), they are immediately replaced. To maintain the desired mean household size and numbers of daily and weekly-monthly contacts, when an individual (A) dies in the model they are immediately replaced by another person, who is a member of the same household and has the same daily and weekly-monthly contacts. To prevent the bias that would result if uninfected people were continually introduced into parts of the network where infection risk is high, if the person dies of TB, the replacement person (B) is given the characteristics of another person (C) chosen at random from the model population. B is therefore given the household, daily and weekly-monthly contacts of person A, and the age and HIV and TB disease characteristics of person C. Person C is then replaced, and becomes a new person, while retaining their household, daily and weekly-monthly contacts.

As people are born into the model at age 15, some will already be infected with *Mtb* and/or HIV. Newly created people are HIV positive with probability *prop_15_HIV_pos* and have a latent TB infection with probability *prop_15_latent.* An input parameter *prop_15_recent_latent_annual* gives the proportion of newly created people that are in each of the five early latent subgroups (<1 year, 1-2 year, 2-3 years, 3-4 years, and 4-5 years). The remainder of newly created people with latent infections have late latent infections.

HIV

A proportion of people in the model, *prop_15_HIV_pos*, are created HIV positive. All other people in the model become HIV positive each month with probability *HIV_infection_rate*. HIV increases the rate at which latently infected people develop disease, reduces the protection against reinfection from being latently infected, reduces the probability that someone developing disease will be smear positive, increases the rate of converting from smear negative to smear positive disease, reduces self-cure rates, and increases TB mortality rates. People with HIV in the model can have different rates of starting and dropping out of treatment, and different reductions in mortality when receiving TB treatment.

Tuberculosis

Disease states

Each individual in the model is in one of eight TB states, as shown in Figure S2

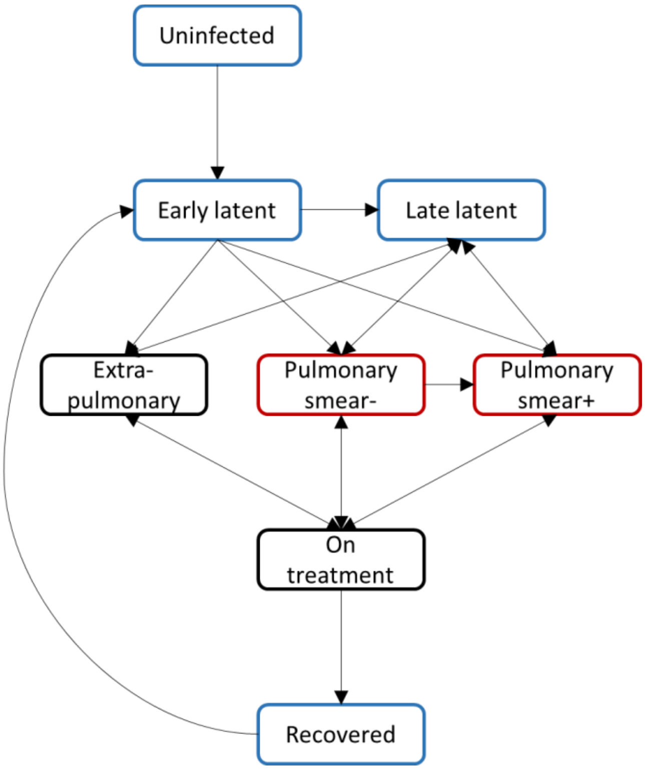

Figure S2. Simulated TB states. Red boxes indicate that the state is infectious, and blue that the state is susceptible to (re)infection.

Disease progression

The rate of developing disease following infection depends on an individual's time since infection, HIV status, and *susceptibility* parameter. The probability of developing disease each month for an individual with an early or late latent infection is:

*1 - exp(- develop_tb_stage_rate * (prop_sus_var_infection + (1 - prop_sus_var_infection) * susceptibility) * HIV_risk_develop_disease_adjustment)*

Where:

- *develop_tb_stage_rate* is the baseline rate of developing disease per month by time since infection:
    - <1 year (early latent): *develop_tb_y1_rate*
    - 1-2 years (early latent): *develop_tb_y2_rate*
    - 2-3 years (early latent): *develop_tb_y3_rate*

    - 3-4 years (early latent): *develop_tb_y4_rate*
    - 4-5 years (early latent): *develop_tb_y5_rate*
    - 5+ years (late latent): *develop_tb_reactivation_rate*
- *(prop_sus_var_infection + (1 - prop_sus_var_infection) * susceptibility)* adjusts the rate of disease development, taking into account the relative susceptibility of the individual, and the proportion of that relative susceptibility that acts on the probability of disease development (as opposed to the probability of infection)
- *HIV_risk_develop_disease_adjustment* is equal to 1 if the individual is HIV negative, and *HIV_increased_risk_develop_disease* if they are HIV positive.

Upon developing disease, HIV negative (HIV positive) people develop extrapulmonary disease with probability *prop_extrapulmonary_HIVneg (prop_extrapulmonary_HIVpos)*, and pulmonary disease with probability *1 - prop_extrapulmonary_HIVneg* (*1 - prop_extrapulmonary_HIVpos*). If pulmonary, they develop smear positive disease with probability *prop_pul_smearpos_HIVneg (prop_pul_smearpos_HIVpos)*, and smear negative disease with probability *1 - prop_pul_smearpos_HIVneg (1 - prop_pul_smearpos_HIVpos)*.

Individuals with smear negative disease convert to smear positive disease each month with probability *develop_smearpos_rate_HIVneg* if HIV negative, and *develop_smearpos_rate_HIVpos* if HIV positive.

Individuals with pulmonary disease self-cure each month with probability *self_cure_rate* if HIV negative, and *self_cure_rate * reduced_self_cure_HIVpos* if HIV positive. Upon self-cure, individuals enter the late latent stage.

Treatment

Individuals with TB start treatment each month with probability *treatment_rate_HIVneg* if HIV negative, and *treatment_rate_HIVpos* if HIV positive, and drop out of treatment each month with probability *TB_treatment_dropout_rate_HIVneg* if HIV negative, and *TB_treatment_dropout_rate_HIVpos* if HIV positive. Upon dropping out of treatment, they return to the disease stage they were in before starting treatment (extrapulmonary, pulmonary smear negative, or pulmonary smear positive).

After a continuous six-month period on treatment, individuals enter the recovered stage.

Mortality

TB mortality rates in the model depend on disease, HIV status, and whether someone is receiving treatment or not. Among people not on treatment, the monthly mortality rate is *TB_mortality_rate_*

smearpos_HIVneg (*TB_mortality_rate_smearpos_HIVpos*) for HIV negative (HIV positive) people with extrapulmonary or pulmonary smear positive disease, and *TB_mortality_rate_smearneg_HIVneg* (*TB_mortality_rate_smearneg_HIVpos*) for HIV negative (HIV positive) people with pulmonary smear negative disease.

When on treatment, mortality rates are adjusted by *relative_TB_mortality_treatment_HIVneg* and *relative_TB_mortality_treatment_HIVneg* for HIV negative and HIV positive people respectively.

Variation in susceptibility and infectiousness

Each individual in the model has a susceptibility parameter and an infectiousness parameter. These are selected at birth from gamma distributions with mean 1 and variance *susceptibility_var* and *infectiousness_var* respectively. It is assumed that there is no clustering of susceptibility or infectiousness in households.

The *susceptibility* parameter is assumed to incorporate the effects of all risk factors that have an effect on the risk of infection and/or disease development, with the exception of HIV, which is explicitly simulated. A parameter, *prop_sus_var_thr_infection*, controls the proportion of the variation in susceptibility that acts through altering the risk of infection (*prop_sus_var_thr_infection*), and the proportion that acts through altering the risk of disease development (*1 - prop_sus_var_thr_infection*).

The *infectiousness* parameter is assumed to incorporate the effects of all risk factors that have an effect on the infectiousness of a person with TB, with the exception of whether the disease is pulmonary smear positive, pulmonary smear negative, or extrapulmonary. If a person has extrapulmonary TB, it is assumed that they cannot transmit *Mtb.* If a person has pulmonary smear negative disease, then their infectiousness is assumed to be reduced by *reduced_trans_smear_neg*. The *infectiousness* parameter and the disease status of an individual are combined to give each simulated individual with TB an *adjusted_infectiousness*, as follows:

- If extrapulmonary, *adjusted_infectiousness = 0*
- If smear positive, *adjusted_infectiousness* = *infectiousness*
- If smear negative *adjusted_infectiousness* = *infectiousness* * *reduced_trans_smear_neg*

Transmission

Each day that a meeting occurs between (A) a person with pulmonary TB and (B) a person who is susceptible (uninfected, early latent, late latent, recovered), there is a probability of transmission. The probability is equal to:

*1 - exp(- transmission_prob * (1 - prop_sus_var_infection + prop_sus_var_infection * susceptibility of (B)) * adjusted_infectiousness of (A)* contact_duration * contact_intensity_adjustment * reinfection_adjustment)*

Where:

- Transmission probability gives the baseline probability of TB transmission in the model, per minute of meeting time
- *(1 - prop_sus_var_infection + prop_sus_var_infection * susceptibility of (B))* adjusts the probability of transmission, taking into account the relative susceptibility of B, and the proportion of that relative susceptibility that acts on the probability of infection (as opposed to the probability of developing disease)
- *adjusted_infectiousness of (A)* is the relative infectiousness of A, taking into account their disease state (extrapulmonary, pulmonary smear negative, or pulmonary smear positive), and their innate infectiousness
- *contact_duration* is the mean duration of meetingd in minutes of the relevant meeting type (household, daily, weekly-monthly, non-repeated close, or non-repeated casual)
- *contact_intensity_adjustment* is set equal to one if the contact type is household, daily, weekly-monthly, or non-repeated close, and *reduced_transmission_non-repeated casual* if the contact type is non-repeated casual
- *reinfection_adjustment* is equal to 1 if B is uninfected, *reduced_transmission_reinfection_ HIV-* if they are latent or recovered and HIV-, and *reduced_transmission_reinfection_HIV+* is they are latent or recovered and HIV+.

Epidemiological input parameter table

Table S1. Model input parameters and their plausible ranges

Name	Description	Range	Source
transmission_prob	Baseline probability of *Mtb* transmission per minute meeting time	0-1	Varied freely to fit to data
reduced_transmission_smearneg	Lower transmission probability with smear negative disease, relative to smear positive	0.1-0.37	Houben[2]
transmission_prob_notalk_weight	Transmission probability per minute for contacts who do not talk (ie non-repeated casual contacts), relative to contacts who do talk	Baseline: 1. Varied in sensitivity analysis	NA
reinfection_relative_risk_HIVneg	Reduced probability of transmission to people with latent infections, relative to uninfected people (HIV negative)	0.1-0.63	Houben[2]
reinfection_relative_risk_HIVpos	Reduced probability of transmission to people with latent infections, relative to uninfected people (HIV positive)	0.5-1	Dowdy and Chaisson[3]
prop_sus_var_infection	The proportion of variation in susceptibility that acts through altering susceptibility to infection (as opposed to disease progression)	Baseline: 0.25. Varied in sensitivity analysis	NA
susceptibility_var	The between-individual variance in susceptibility	≥0	Varied freely to fit to data
infectiousness_var	The between-individual variance in infectiousness	≥0	Varied freely to fit to data
self_cure_rate	The rate of self-cure per month for HIV negative people	0.0087-0.024	Houben[2]
reduced_self_cure_HIVpos	The rate of self-cure per month for HIV positive people, relative to the rate in HIV negative people	0.25	Estimated from Menzies *et al*[4]

TB_mortality_rate_ smearpos_HIVneg	Rate of mortality per month from smear positive pulmonary or extrapulmonary TB for HIV negative people	0.018-0.043	Houben[2]
TB_mortality_rate_ smearpos_HIVpos	Rate of mortality per month from smear positive pulmonary or extrapulmonary TB for HIV positive people	> TB_mortality_rate_ smearpos_HIVneg	Houben[2]
TB_mortality_rate_ smearneg_HIVneg	Rate of mortality per month from smear negative pulmonary TB for HIV negative people	0.016-0.024	Houben[2]
TB_mortality_rate_ smearneg_HIVpos	Rate of mortality per month from smear negative pulmonary TB for HIV positive people	> TB_mortality_rate_ smearneg_HIVneg, < TB_mortality_rate_ smearpos_HIVpos	Houben[2]
relative_TB_mortality_ treatment_HIVneg	Rate of TB mortality per month when on treatment for HIV negative people, relative to rate in people not on treatment with the same disease type	>0	Varied freely to fit to data
relative _TB_mortality_ treatment_HIVpos	Rate of TB mortality per month when on treatment for HIV positive people, relative to rate in people not on treatment with the same disease type	>0	Varied freely to fit to data
TB_treatment_dropout_ rate_HIVneg	Rate of dropping out of TB treatment per month for HIV negative people	0-1	Varied freely to fit to data
TB_treatment_dropout_ rate_HIVpos	Rate of dropping out of TB treatment per month for HIV positive people	0-1	Varied freely to fit to data
treatment_rate_extra_ HIVneg	Rate of starting treatment per month for HIV negative people with extrapulmonary TB	0-1	Varied freely to fit to data
treatment_rate_extra_ HIVpos	Rate of starting treatment per month for HIV positive people with extrapulmonary TB	0-1	Varied freely to fit to data
treatment_rate_ smearneg_HIVneg	Rate of starting treatment per month for HIV negative people with smear negative pulmonary TB	0-1	Varied freely to fit to data

treatment_rate_ smearneg_HIVpos	Rate of starting treatment per month for HIV positive people with smear negative pulmonary TB	0-1	Varied freely to fit to data
treatment_rate_smearpos_ HIVneg	Rate of starting treatment per month for HIV negative people with smear positive pulmonary TB	0-1	Varied freely to fit to data
treatment_rate_smearpos_ HIVpos	Rate of starting treatment per month for HIV positive people with smear positive pulmonary TB	0-1	Varied freely to fit to data
develop_tb_y1_rate	Baseline rate of developing TB per month for HIV negative people during the first year following infection (before adjustment for individual susceptibility to disease progression)	0.0068-0.0083	Kasaie *et al*[5]
develop_tb_y2_rate	Baseline rate of developing TB per month for HIV negative people during the second year following infection (before adjustment for individual susceptibility to disease progression)	0.0027-0.0033	Kasaie *et al*[5]
develop_tb_y3_rate	Baseline rate of developing TB per month for HIV negative people during the third year following infection (before adjustment for individual susceptibility to disease progression)	0.00084-0.00103	Kasaie *et al*[5]
develop_tb_y4_rate	Baseline rate of developing TB per month for HIV negative people during the fourth year following infection (before adjustment for individual susceptibility to disease progression)	0.00056-0.00068	Kasaie *et al*[5]
develop_tb_y5_rate	Baseline rate of developing TB per month for HIV negative people during the fifth year following infection (before adjustment for individual susceptibility to disease progression)	0.00015-0.00018	Kasaie *et al*[5]
develop_tb_reactivation_ rate	Baseline rate of developing TB per month for HIV negative people who have been infected for more than 5 years (late latent) (before adjustment for individual susceptibility to disease progression)	1.67×10^{-5}-8.34×10^{-5}	Kasaie *et al*[5]
HIV_increased_risk_ develop_disease	Rate of developing disease if HIV positive, relative to rate if HIV negative with the same duration of infection	>1	Varied freely to fit to data
prop_extrapulmonary_ HIVneg	Proportion of HIV negative people developing disease who develop extrapulmonary disease	0.05	Murray and Salomon[6]

prop_pul_smearpos_ HIVneg	Proportion of HIV negative people who develop pulmonary disease who initially develop smear positive disease	0.45	Murray and Salomon[6]
prop_extrapulmonary_ HIVpos	Proportion of HIV positive people developing disease who develop extrapulmonary disease	0.05	Murray and Salomon[6]
prop_pul_smearpos_ HIVpos	Proportion of HIV positive people who develop pulmonary disease who initially develop smear positive disease	0.35	Murray and Salomon[6]
develop_smearpos_rate_ HIVneg	Rate of converting from smear negative to smear positive pulmonary disease per month, if HIV negative	0.00059-0.0025	Houben[2]
develop_smearpos_rate_ HIVpos	Rate of converting from smear negative to smear positive pulmonary disease per month, if HIV positive	0.0013-0.0025	Houben[2]
initial_prop_HIV_pos	Proportion of people made HIV positive during model initialisation	0.192	Same as fitting HIV prevalence
HIV_infection_rate	Rate of becoming HIV positive, per month	0-1	Varied freely to fit to data
prop_15_HIV_pos	Proportion of people 'born' into the model at age 15 who are HIV positive	0.06	Middelkoop *et al*[1]
prop_15_latent	Proportion of people 'born' into the model at age 15 who have latent infections	0.475	Middelkoop *et al*[1]
prop_15_recent_latent_ annual	Proportion of people 'born' into the model at age 15 who are in each of the five early latent infection categories (<1y, 1-2y, 2-3y, 3-4y, and 4-5y)	0.03	Middelkoop *et al*[1]
weekly_pool_size	Input used to determine the mean total number of weekly-monthly contacts each simulated individual should have	Baseline: 5. Varied in sensitivity analysis	NA
cluster_coeff	Clustering co-efficient for repeated contact	Baseline: 0.2. Varied in sensitivity analysis	NA

Model fitting outputs and plausible ranges

Table S2. Model fitting outputs and their plausible ranges

Description	Plausible range	Source
Estimated incidence (all forms) per 100 000 population	539-1190	WHO Global report[7]
Estimated incidence of TB cases who are HIV-positive per 100 000 population	303-680	WHO Global report[7]
Estimated mortality of TB cases (all forms, excluding HIV) per 100,000 population	39-53	WHO Global report[7]
Estimated mortality of TB cases who are HIV-positive, per 100,000 population	50-256	WHO Global report[7]
HIV negative treatment success (proportion)	0.72-0.88	The Electronic Tuberculosis Register[8]
HIV positive treatment success (proportion)	0.68-0.84	The Electronic Tuberculosis Register[8]
Proportion HIV negative dying while on TB treatment	>0.044	The Electronic Tuberculosis Register[8]
Proportion HIV positive dying while on TB treatment	>0.073	The Electronic Tuberculosis Register[8]
Estimated TB case fatality ratio	0.22-0.42	WHO Global report[7]
Case detection rate (all forms), percent	0.44-0.98	WHO Global report[7]
HIV prevalence	0.18-0.20	UNAIDS 2015 estimate[9]
Proportion of people starting treatment who are HIV positive	0.51-0.62	WHO Global report[7]

Additional methods

Sensitivity analyses

In addition to varying the probability of transmission for contacts who did not talk, relative to the probability for other contact types, we conducted a number of other sensitivity analyses:

- Varying the mean total number of weekly-monthly contacts each simulated individual has (*weekly_pool_size*) from its default value of 5 to 1.17 and 10.
- Varying the proportion of variation in susceptibility that acts through altering the susceptibility to infection (as opposed to susceptibility to disease progression) (*prop_sus_var_infection*) from its default value of 0.25 to 0.1 and 0.75.
- In the model, the amount of variation between people in susceptibility (*susceptibility_var*) and the amount of variation between people in infectiousness (*infectiousness_var*) are set equal. We explored the effects of simulating no variation in susceptibility (*susceptibility_var* = 0), and altering the amount of variation in infectiousness only; and of simulating twice as much variation in susceptibility as infectiousness (*infectiousness_var = 0.5 * susceptibility_var*).
- Varying the clustering coefficient for repeated contacts (*cluster_coeff*) from its default value of 0.2 to 0 and 0.4.
- Assuming that 20% of casual contacts were repeated.

All sensitivity analyses listed above were conducted using the high non-repeated casual transmission risk scenario, fitted to the best estimate of k ($k = 0.147$).

Additional results

Fitted input parameter values

Table S3. Model input parameter values in the fitted scenarios

Parameter	High casual transmission				Medium casual transmission				Low casual transmission			
	No additional variation	k = 0.015	k = 0.074	k = 0.32	No additional variation	k = 0.015	k = 0.074	k = 0.32	No additional variation	k = 0.015	k = 0.074	k = 0.32
transmission_prob	1.83E-05	2.07E-05	2.92E-05	1.58E-05	3.06E-05	4.06E-05	5.84E-05	3.10E-05	5.25E-05	9.51E-05	1.42E-04	6.56E-05
reduced_transmission_smearneg	0.22	0.22	0.22	0.22	0.22	0.22	0.22	0.22	0.22	0.22	0.22	0.22
transmission_prob_notalk_weight	1	1	1	1	0.5	0.5	0.5	0.5	0.2	0.2	0.2	0.2
reinfection_relative_risk_HIVneg	0.35	0.35	0.35	0.35	0.35	0.35	0.35	0.35	0.35	0.35	0.35	0.35
reinfection_relative_risk_HIVpos	0.75	0.75	0.75	0.75	0.75	0.75	0.75	0.75	0.75	0.75	0.75	0.75
prop_sus_var_infection	0.25	0.25	0.25	0.25	0.25	0.25	0.25	0.25	0.25	0.25	0.25	0.25
susceptibility_var	0	5.3	13.8	1.04	0	6.48	16	1.75	0	0	0	0
infectiousness_var	0	5.3	13.8	1.04	0	6.48	16	1.75	0	0	0	0
self_cure_rate	0.0237	0.0237	0.0237	0.0237	0.0237	0.0237	0.0237	0.0237	0.0237	0.0237	0.0237	0.0237

reduced_self_cure_ HIVpos	0.25	0.25	0.25	0.25	0.25	0.25	0.25	0.25	0.25	0.25	0.25	0.25
TB_mortality_rate_ smearpos_HIVneg	0.0184	0.0184	0.0184	0.0184	0.0184	0.0184	0.0184	0.0184	0.0184	0.0184	0.0184	0.0184
TB_mortality_rate_ smearpos_HIVpos	0.0874	0.104	0.104	0.104	0.0874	0.104	0.104	0.104	0.0874	0.104	0.104	0.104
TB_mortality_rate_ smearneg_HIVneg	0.0164	0.0164	0.0164	0.0164	0.0164	0.0164	0.0164	0.0164	0.0164	0.0164	0.0164	0.0164
TB_mortality_rate_ smearneg_HIVpos	0.0463	0.0913	0.0913	0.0913	0.0463	0.0913	0.0913	0.0913	0.0463	0.0913	0.0913	0.0913
relative_TB_mortality_ treatment_HIVneg	0.486	0.486	0.486	0.486	0.486	0.486	0.486	0.486	0.486	0.486	0.486	0.486
relative _TB_mortality_ treatment_HIVpos	0.237	0.14	0.14	0.14	0.237	0.14	0.14	0.14	0.237	0.14	0.14	0.14
TB_treatment_dropout_ rate_HIVneg	0.038	0.038	0.038	0.038	0.038	0.038	0.038	0.038	0.038	0.038	0.038	0.038
TB_treatment_dropout_ rate_HIVpos	0.038	0.038	0.038	0.038	0.038	0.038	0.038	0.038	0.038	0.038	0.038	0.038
treatment_rate_extra_ HIVneg	0.0603	0.0603	0.0603	0.0603	0.0603	0.0603	0.0603	0.0603	0.0603	0.0603	0.0603	0.0603
treatment_rate_extra_ HIVpos	0.166	0.166	0.166	0.166	0.166	0.166	0.166	0.166	0.166	0.166	0.166	0.166
treatment_rate_ smearneg_HIVneg	0.0966	0.0966	0.0966	0.0966	0.0966	0.0966	0.0966	0.0966	0.0966	0.0966	0.0966	0.0966

treatment_rate_ smearneg_HIVpos	0.149	0.149	0.149	0.149	0.149	0.149	0.149	0.149	0.149	0.149	0.149	0.149
treatment_rate_ smearpos_HIVneg	0.0966	0.0966	0.0966	0.0966	0.0966	0.0966	0.0966	0.0966	0.0966	0.0966	0.0966	0.0966
treatment_rate_ smearpos_HIVpos	0.149	0.149	0.149	0.149	0.149	0.149	0.149	0.149	0.149	0.149	0.149	0.149
develop_tb_y1_rate	7.52E-03	7.52E-03	7.52E-03	7.52E-03	7.52E-03	7.52E-03	7.52E-03	7.52E-03	7.52E-03	7.52E-03	7.52E-03	7.52E-03
develop_tb_y2_rate	3.01E-03	3.01E-03	3.01E-03	3.01E-03	3.01E-03	3.01E-03	3.01E-03	3.01E-03	3.01E-03	3.01E-03	3.01E-03	3.01E-03
develop_tb_y3_rate	9.38E-04	9.38E-04	9.38E-04	9.38E-04	9.38E-04	9.38E-04	9.38E-04	9.38E-04	9.38E-04	9.38E-04	9.38E-04	9.38E-04
develop_tb_y4_rate	6.19E-04	6.19E-04	6.19E-04	6.19E-04	6.19E-04	6.19E-04	6.19E-04	6.19E-04	6.19E-04	6.19E-04	6.19E-04	6.19E-04
develop_tb_y5_rate	1.67E-04	1.67E-04	1.67E-04	1.67E-04	1.67E-04	1.67E-04	1.67E-04	1.67E-04	1.67E-04	1.67E-04	1.67E-04	1.67E-04
develop_tb_reactivation_ rate	4.17E-05	4.17E-05	4.17E-05	4.17E-05	4.17E-05	4.17E-05	4.17E-05	4.17E-05	4.17E-05	4.17E-05	4.17E-05	4.17E-05
HIV_increased_risk_ develop_disease	7.84	20	20	20	7.84	20	20	20	7.84	20	20	20
prop_extrapulmonary_ HIVneg	0.05	0.05	0.05	0.05	0.05	0.05	0.05	0.05	0.05	0.05	0.05	0.05

prop_pul_smearpos_ HIVneg	0.45	0.45	0.45	0.45	0.45	0.45	0.45	0.45	0.45	0.45	0.45	0.45
prop_extrapulmonary_ HIVpos	0.05	0.05	0.05	0.05	0.05	0.05	0.05	0.05	0.05	0.05	0.05	0.05
prop_pul_smearpos_ HIVpos	0.35	0.35	0.35	0.35	0.35	0.35	0.35	0.35	0.35	0.35	0.35	0.35
develop_smearpos_rate_ HIVneg	1.26E-03	1.26E-03	1.26E-03	1.26E-03	1.26E-03	1.26E-03	1.26E-03	1.26E-03	1.26E-03	1.26E-03	1.26E-03	1.26E-03
develop_smearpos_rate_ HIVpos	1.89E-03	1.89E-03	1.89E-03	1.89E-03	1.89E-03	1.89E-03	1.89E-03	1.89E-03	1.89E-03	1.89E-03	1.89E-03	1.89E-03
initial_prop_HIV_pos	0.192	0.192	0.192	0.192	0.192	0.192	0.192	0.192	0.192	0.192	0.192	0.192
HIV_infection_rate	6.72E-04	7.35E-04	7.35E-04	7.35E-04	6.72E-04	7.35E-04	7.35E-04	7.35E-04	6.72E-04	7.35E-04	7.35E-04	7.35E-04
prop_15_HIV_pos	0.06	0.06	0.06	0.06	0.06	0.06	0.06	0.06	0.06	0.06	0.06	0.06
prop_15_latent	0.475	0.475	0.475	0.475	0.475	0.475	0.475	0.475	0.475	0.475	0.475	0.475
prop_15_recent_latent_ annual	0.03	0.03	0.03	0.03	0.03	0.03	0.03	0.03	0.03	0.03	0.03	0.03
weekly_pool_size	5	5	5	5	5	5	5	5	5	5	5	5
cluster_coeff	0.2	0.2	0.2	0.2	0.2	0.2	0.2	0.2	0.2	0.2	0.2	0.2

Table S4. Model input parameter values in the sensitivity analysis scenarios.

Parameter	weekly_ pool_ size = 1.17	weekly_ pool_ size = 10	prop_ sus_var_ infection = 0.1	prop_ sus_var_ infection = 0.75	susceptibility_ var = 0	infectiousness_ var = 0.5 * susceptibility_ var	cluster_coeff = 0	cluster_coeff = 0.4	20% of casual contacts regular
transmission_prob	2.07E-05	2.01E-05	2.93E-05	2.07E-05	1.88E-05	2.32E-05	2.03E-05	2.07E-05	2.43E-05
reduced_transmission_ smearneg	0.22	0.22	0.22	0.22	0.22	0.22	0.22	0.22	0.22
transmission_prob_ notalk_weight	1	1	1	1	1	1	1	1	1
reinfection_ relative_risk_HIVneg	0.35	0.35	0.35	0.35	0.35	0.35	0.35	0.35	0.35
reinfection_relative_ risk_HIVpos	0.75	0.75	0.75	0.75	0.75	0.75	0.75	0.75	0.75
prop_sus_var_infection	0.25	0.25	0.1	0.75	0.25	0.75	0.25	0.25	0.25
susceptibility_var	5.3	5.2	5.3	5.3	0	10.4	5.3	5.3	5.8
infectiousness_var	5.3	5.2	5.3	5.3	5.5	5.2	5.3	5.3	5.8
self_cure_rate	0.0237	0.0237	0.0237	0.0237	0.0237	0.0237	0.0237	0.0237	0.0237
reduced_self_cure_ HIVpos	0.25	0.25	0.25	0.25	0.25	0.25	0.25	0.25	0.25

TB_mortality_rate_ smearpos_HIVneg	0.0184	0.0184	0.0184	0.0184	0.0184	0.0184	0.0184	0.0184	0.0184
TB_mortality_rate_ smearpos_HIVpos	0.104	0.104	0.104	0.104	0.104	0.104	0.104	0.104	0.104
TB_mortality_rate_ smearneg_HIVneg	0.0164	0.0164	0.0164	0.0164	0.0164	0.0164	0.0164	0.0164	0.0164
TB_mortality_rate_ smearneg_HIVpos	0.0913	0.0913	0.0913	0.0913	0.0913	0.0913	0.0913	0.0913	0.0913
relative_TB_mortality_ treatment_HIVneg	0.486	0.486	0.486	0.486	0.486	0.486	0.486	0.486	0.486
relative _TB_mortality_ treatment_HIVpos	0.14	0.14	0.14	0.14	0.14	0.14	0.14	0.14	0.14
TB_treatment_ dropout_rate_HIVneg	0.038	0.038	0.038	0.038	0.038	0.038	0.038	0.038	0.038
TB_treatment_ dropout_rate_HIVpos	0.038	0.038	0.038	0.038	0.038	0.038	0.038	0.038	0.038
treatment_rate_extra_ HIVneg	0.0603	0.0603	0.0603	0.0603	0.0603	0.0603	0.0603	0.0603	0.0603
treatment_rate_extra_ HIVpos	0.166	0.166	0.166	0.166	0.166	0.166	0.166	0.166	0.166
treatment_rate_ smearneg_HIVneg	0.0966	0.0966	0.0966	0.0966	0.0966	0.0966	0.0966	0.0966	0.0966

treatment_rate_ smearneg_HIVpos	0.149	0.149	0.149	0.149	0.149	0.149	0.149	0.149	0.149
treatment_rate_ smearpos_HIVneg	0.0966	0.0966	0.0966	0.0966	0.0966	0.0966	0.0966	0.0966	0.0966
treatment_rate_ smearpos_HIVpos	0.149	0.149	0.149	0.149	0.149	0.149	0.149	0.149	0.149
develop_tb_y1_rate	7.52E-03	7.52E-03	7.52E-03	7.52E-03	7.52E-03	7.52E-03	7.52E-03	7.52E-03	7.52E-03
develop_tb_y2_rate	3.01E-03	3.01E-03	3.01E-03	3.01E-03	3.01E-03	3.01E-03	3.01E-03	3.01E-03	3.01E-03
develop_tb_y3_rate	9.38E-04	9.38E-04	9.38E-04	9.38E-04	9.38E-04	9.38E-04	9.38E-04	9.38E-04	9.38E-04
develop_tb_y4_rate	6.19E-04	6.19E-04	6.19E-04	6.19E-04	6.19E-04	6.19E-04	6.19E-04	6.19E-04	6.19E-04
develop_tb_y5_rate	1.67E-04	1.67E-04	1.67E-04	1.67E-04	1.67E-04	1.67E-04	1.67E-04	1.67E-04	1.67E-04
develop_tb_ reactivation_rate	4.17E-05	4.17E-05	4.17E-05	4.17E-05	4.17E-05	4.17E-05	4.17E-05	4.17E-05	4.17E-05
HIV_increased_risk_ develop_disease	20	20	20	20	12	20	20	20	20
prop_extrapulmonary_ HIVneg	0.05	0.05	0.05	0.05	0.05	0.05	0.05	0.05	0.05
prop_pul_smearpos_ HIVneg	0.45	0.45	0.45	0.45	0.45	0.45	0.45	0.45	0.45
prop_extrapulmonary_ HIVpos	0.05	0.05	0.05	0.05	0.05	0.05	0.05	0.05	0.05

prop_pul_smearpos_ HIVpos	0.35	0.35	0.35	0.35	0.35	0.35	0.35	0.35	0.35
develop_smearpos_ rate_HIVneg	1.26E-03	1.26E-03	1.26E-03	1.26E-03	1.26E-03	1.26E-03	1.26E-03	1.26E-03	1.26E-03
develop_smearpos_ rate_HIVpos	1.89E-03	1.89E-03	1.89E-03	1.89E-03	1.89E-03	1.89E-03	1.89E-03	1.89E-03	1.89E-03
initial_prop_HIV_pos	0.192	0.192	0.192	0.192	0.192	0.192	0.192	0.192	0.192
HIV_infection_rate	7.35E-04	7.35E-04	7.35E-04	7.35E-04	7.35E-04	7.35E-04	7.35E-04	7.35E-04	7.35E-04
prop_15_HIV_pos	0.06	0.06	0.06	0.06	0.06	0.06	0.06	0.06	0.06
prop_15_latent	0.475	0.475	0.475	0.475	0.475	0.475	0.475	0.475	0.475
prop_15_recent_latent_ annual	0.03	0.03	0.03	0.03	0.03	0.03	0.03	0.03	0.03
weekly_pool_size	1.17	10	5	5	5	5	5	5	5
cluster_coeff	0.2	0.2	0.2	0.2	0.2	0.2	0	0.4	0.2

Sensitivity analyses

Fit to Data

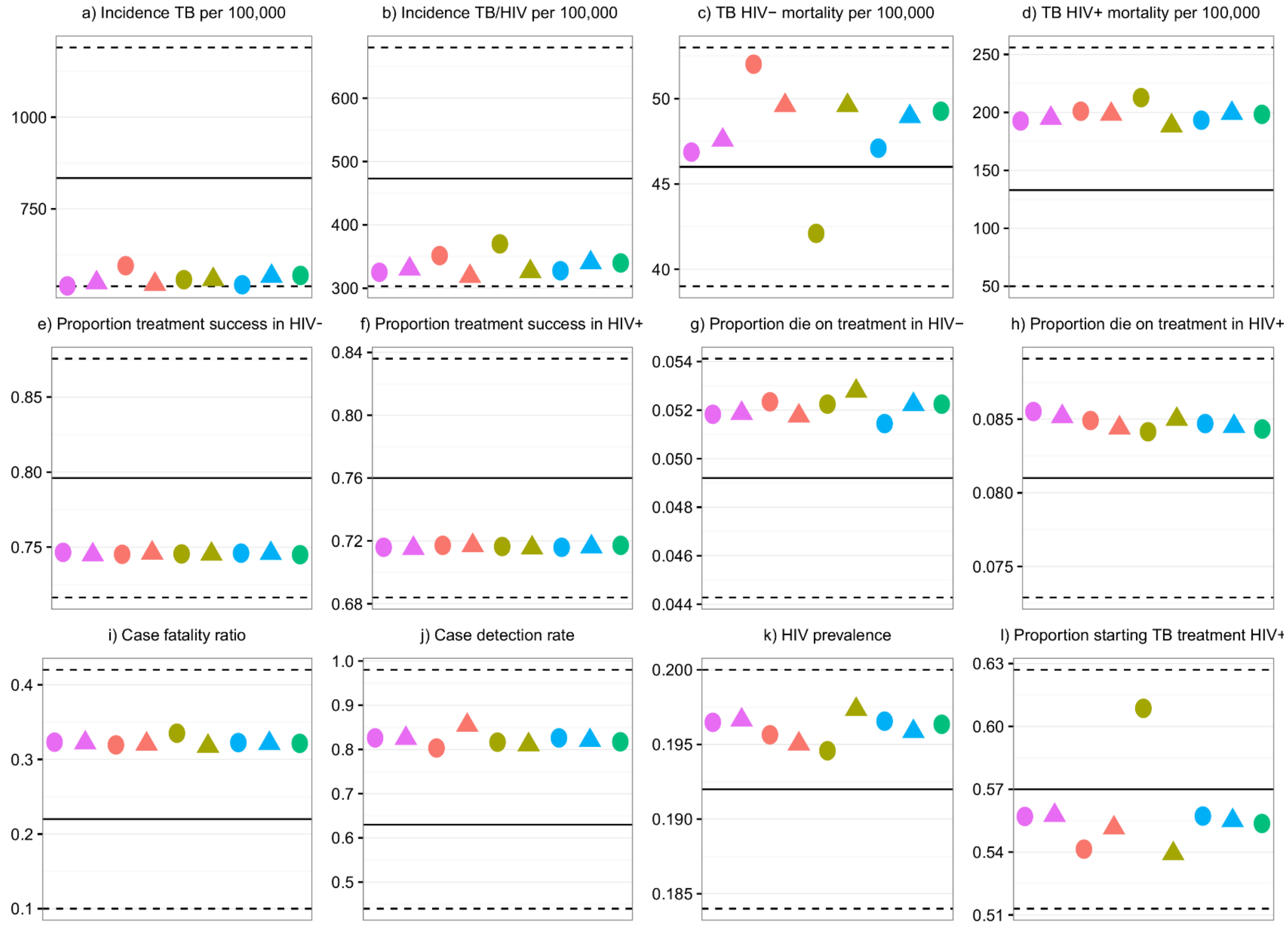

Figure S3. Model fit to data. The solid horizontal lines indicate the best estimates of the output values. The dashed horizontal lines indicate the minimum and maximum of the output plausible ranges. Pink circles and triangles show the *weekly_pool_size = 1.17* and *weekly_pool_size = 10* scenarios respectively, red circles and triangles show the *prop_sus_var_infection = 0.1* and *prop_sus_var_infection = 0.75* scenarios respectively, yellow circles and triangles show the *susceptibility_var = 0* and *infectiousness_var = 0.5 * susceptibility_var* scenarios respectively, blue circles and triangles show the *cluster_coeff = 0* and *cluster_coeff = 0.4* scenarios respectively, and green circles show 20% of casual contacts regular.

Results

There was little variation between the baseline scenario and the sensitivity analyses scenarios in the proportion of transmission by household (12.6% in baseline, 11.6%-13.4% in sensitivity analyses), repeated (8.16% in baseline, 5.97%-12.5% in sensitivity analyses), or non-repeated (79.3% in baseline, 74.6%-81.3% in sensitivity analyses) contacts (Table S5). There was also little variation in the proportion of transmission by the most highly transmitting 20%, 10%, 5%, 2%, or 1% of people with pulmonary TB (Figure S4).

Table S5. Proportion of TB cases resulting from transmission between household, repeated, and non-repeated contacts, in the baseline and sensitivity analysis scenarios

		**Baseline**[1]	***weekly_ pool_size = 1.17***	***weekly_ pool_size = 10***	***prop_sus_ var_ infection = 0.1***	***prop_sus_ var_ infection = 0.75***	***susceptibility_ var = 0***	***infectiousness_var = 0.5 * susceptibility_var***	***cluster_ coeff = 0***	***cluster_ coeff = 0.4***	***20% of casual contacts regular***
**Household**		12.6%	12.7%	12.6%	11.6%	12.8%	13.4%	11.9%	12.6%	12.5%	12.9%
**Repeated**	*Daily*	2.51%	2.45%	2.54%	2.33%	2.51%	2.68%	2.40%	2.54%	2.50%	3.72%
	Weekly-monthly	5.65%	3.52%	6.49%	5.62%	5.59%	5.65%	5.66%	5.68%	5.62%	8.77%
	Overall	8.16%	5.97%	9.03%	7.95%	8.11%	8.33%	8.06%	8.22%	8.13%	12.5%
**Non-repeated**	*Non-repeated close*	1.06%	1.08%	1.03%	1.06%	1.05%	1.05%	1.07%	1.07%	1.04%	1.01%
	Casual	78.2%	80.2%	77.3%	79.3%	78.0%	77.2%	78.9%	78.1%	78.3%	73.6%
	Overall	79.3%	81.3%	78.4%	80.4%	79.1%	78.2%	80.0%	79.2%	79.4%	74.6%

[1]Baseline scenario with high non-repeated casual transmission risk

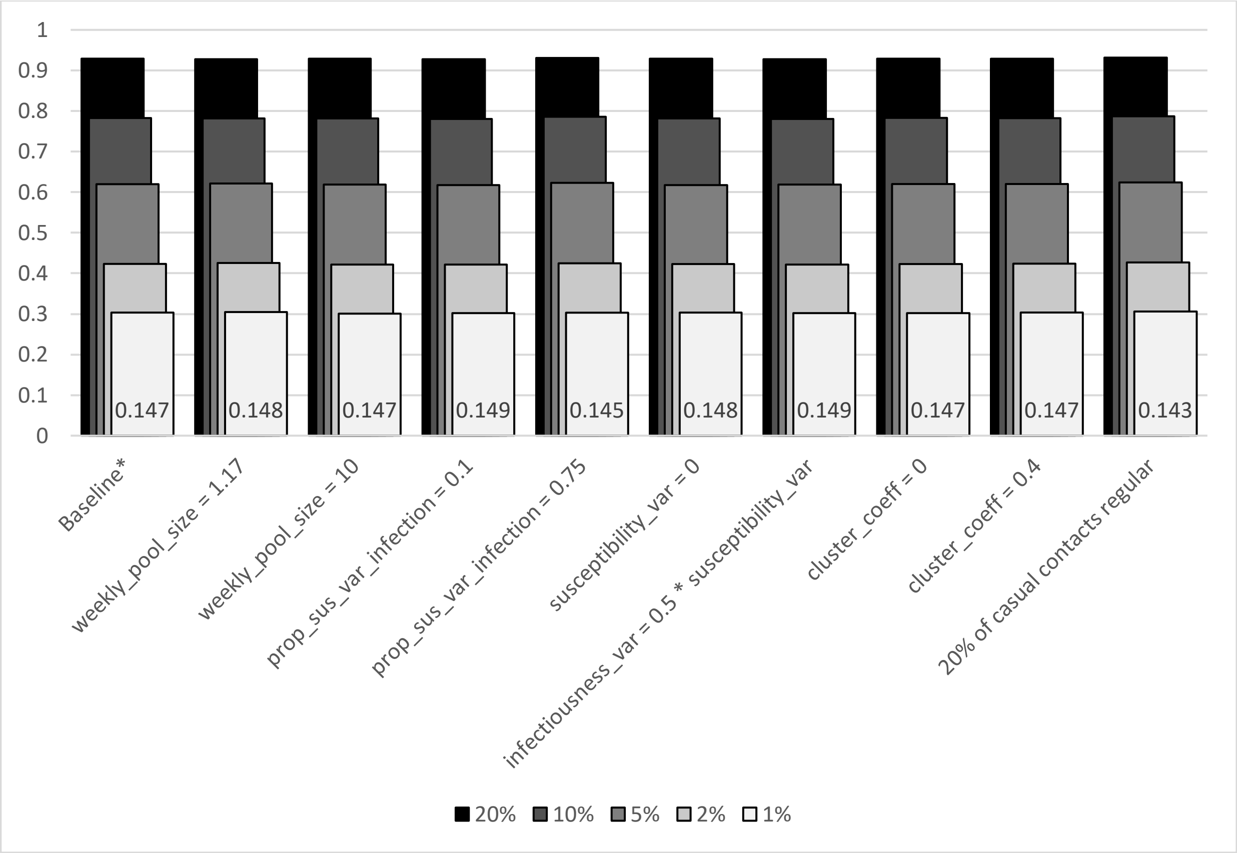

Figure S4. Proportion of transmission resulting in disease by most highly transmitting n% of people with pulmonary TB, in the baseline and sensitivity analysis scenarios. The numbers at the bottom of the bars give the dispersion parameter, k, from fitting a negative binomial distribution to the number of TB cases resulting from transmission by each person with pulmonary TB. * Baseline scenario with high non-repeated casual transmission risk.

References

1. Middelkoop K, Bekker L-G, Morrow C, Lee N, Wood R. Decreasing household contribution to TB transmission with age: a retrospective geographic analysis of young people in a South African township. *BMC Infectious Diseases* **14**, 221 (2014).

2. Houben RMGJ*, et al.* TIME Impact – a new user-friendly tuberculosis (TB) model to inform TB policy decisions. *BMC Medicine* **14**, 56 (2016).

3. Dowdy DW, Chaisson RE. The persistence of tuberculosis in the age of DOTS: reassessing the effect of case detection. *Bulletin of the World Health Organization* **87**, 296-304 (2009).

4. Menzies NA, Cohen T, Lin H-H, Murray M, Salomon JA. Population health impact and cost-effectiveness of tuberculosis diagnosis with Xpert MTB/RIF: a dynamic simulation and economic evaluation. *PloS Med* **9**, e1001347 (2012).

5. Kasaie P, Andrews JR, Kelton WD, Dowdy DW. Timing of Tuberculosis Transmission and the Impact of Household Contact Tracing: An Agent-Based Simulation Model. *American Journal of Respiratory and Critical Care Medicine* **189**, 845-852 (2014).

6. Murray CJ, Salomon JA. *Using mathematical models to evaluate global tuberculosis control strategies*. Harvard Center for Population and Development Studies, Harvard School of Public Health (1998).

7. World Health Organization. Global tuberculosis report 2016. (2016).

8. The electronic tuberculosis register [1/6/16]. Available from: http://www.etrnet.info.

9. UNAIDS. AIDSInfo [23/06/15]. Available from: http://www.unaids.org/en/dataanalysis/datatools/aidsinfo.
